# Research Coproduction: An Underused Pathway to Impact

**DOI:** 10.34172/ijhpm.2024.8461

**Published:** 2024-05-21

**Authors:** Jo Rycroft-Malone, Ian D. Graham, Anita Kothari, Chris McCutcheon

**Affiliations:** ^1^Lancaster University, Lancaster, UK.; ^2^University of Ottawa, Ottawa, ON, Canada.; ^3^University of Western Ontario, London, ON, Canada.; ^4^Ottawa Hospital Research Institute, Ottawa, ON, Canada.

**Keywords:** Research Coproduction, Integrated Knowledge Translation, Implementation

## Abstract

Knowledge translation and implementation science have made many advances in the last two decades. However, research is still not making expedient differences to practice, policy, and service delivery. It is time to evolve our approach to knowledge production and implementation. In this editorial we advance research coproduction as a neglected pathway to impact. Our starting point is that research impact is a function of how research is done and who is involved, arguing that researchers and non-researchers have an equal voice and role to play. We outline principles of coproduction including sharing power, valuing different sources of knowledge and viewpoints, equality, open communication, inclusivity, and mutuality. We consider implications at micro, meso, and macro system levels. In calling for this shift in the way knowledge is produced and applied, we anticipate it leading to inclusive research that more rapidly translates to better, more equitable health and care for all.

## Background

 The continued challenge of research not making a timely difference to policy, service delivery and ultimately to practice provides one motivation for thinking differently about how we do research. The COVID-19 pandemic, and the rise of movements such as Black Lives Matter, #MeToo, Every Child Matters, and Indigenous Lives Matter that have exposed the marginalisation and exclusion of people and groups across all aspects of society provides further motivation. The fields of knowledge translation and implementation science have made great advances, however, there remain persistent gaps between research generation and practice, and therefore between appropriate care and evidence-informed recommendations.^1,2^ As implementation researchers, our starting point is that whether research is used or not is a function of how we create the evidence base. It is time to reflect on and evolve our position on knowledge production and implementation in health and care research.

 In 2010 Best and Holmes conceptualized three generations of thinking about how knowledge to action works.^3^ First, linear models emphasize the transfer of knowledge in a one-way direction from the researcher who produces the evidence (one community) which is disseminated to end users (a different community) and is then used. Second, relationship models incorporate dissemination but then extend to focus on the interactions between people and the development of partnerships and networks as knowledge generation and sharing mechanisms. Third, a systems model builds on linear and relationship conceptualisations by recognizing that diffusion and dissemination processes and actions are embedded and shaped by the context in which they operate, and that as a complex adaptive system these will be dynamic and ever-changing.^4^ Whilst Best and Holmes’ three generations of thinking helpfully illustrate the shift from knowledge transfer to a more distributed and deliberative framing of the challenge, the gaps between research and practice endure. We therefore propose that there needs to be greater attention to conceptualising practice and knowledge production as more synergistic and inextricably linked, meaning that we have a greater chance of creating evidence for real world problems, which can be implemented.

## Research Coproduction as a Strategy to Improve Impact

 Research coproduction is “a model of collaborative research that explicitly responds to knowledge user needs in order to produce research findings that are useful, useable and used”^5^ (p. 1). Research coproduction can occur at project, programme, organisational and system levels, and is theoretically and methodologically pluralistic and can apply to every field of health research. Our starting point is based on a fundamental principle of research coproduction – that all have an equal voice and role to play throughout the research lifecycle,^6^ including implementation.

 Coproduction has been suggested as a necessary action for systems changeand a “potential vehicle for systems thinking in-action”^7^ (p. 2) because of its focus on bringing diverse multiple actors together. There are distinguishing features of research coproduction which amplify its potential for generating evidence-informed solutions that could more rapidly translate into better, more equitable health and care; these features are not yet widely embraced in health research and not in research implementation.

## Distinguishing Features of Research Coproduction

 A research coproduction process is a principle-based and explicitly values-driven approach in which the skills, processes and attitudes required to nurture relationships between knowledge users and researchers are as important as the scientific approach itself. A key distinguishing feature therefore is that it is equity driven where everyone can achieve their full potential for health and well-being. Whilst partnership approaches are not new in the context of participatory research, partnership working framed around a set of agreed principles is an essential ingredient of research coproduction so that it is intentionally and deliberately egalitarian.^8^ These principles include sharing power, valuing different sources of knowledge and viewpoints equally, reciprocity and mutuality, inclusivity, open communication, and attention to practical and financial considerations.^9,10^

 The principles of research coproduction advocate power-sharing, a redefinition of expert/ise and a shift towards equity. However, relinquishing influence and power is notoriously challenging. Orr and Bennett describe the “tricky issues” involved in developing co-operative interactions between members of different communities with distinct interests, priorities and expectations.^11^ There are also power differentials between knowledge systems. Science was a tool of colonization, and Indigenous and local knowledge has often been exploited, subsumed, or marginalised through research — even in “partnered” research.^12^ Coproduction with Indigenous knowledge users and Knowledge Holders demands a commitment to reconciliation and strategies to decolonize the research process.^13^ Given that power typically resides with researchers not knowledge users, achieving an equitable partnership requires scientific humility,^10,14^ and the adoption of practical project management approaches that enable power sharing in governance, roles and activities. We suggest it is researchers who have the greatest capacity and responsibility for adopting a more equitable way of working.

 The foundation of research coproduction is authentic partnership. We advocate an inclusive view of the type of affected and interested parties who may become a partner in the research process, including patients and the public, other decision-makers and so on. Partners come to the research process from different positions and perspectives and have different types of input throughout the research lifecycle, requiring attention to the different roles partners and researchers play and what knowledge and expertise is privileged. As such, providing the space to develop, nurture and sustain meaningful partnerships that allow both disagreement and consensus development is foundational.^15^ We suggest that genuine research coproduction is a function of the quality of the partnership that is cultivated.

 The architectures, or ways in which systems are organised, provide the structures and resources for optimal partnership working. Context is key to determine the processes and outcomes of coproduction where there is often a poor fit with the power hierarchies and incentive structures in the academy.^16^ In particular, the critical determinants of an equitable research coproduction system’s architecture include the role of funders in supporting projects/programmes/partnerships and incentivising behaviour change,^17^ the reward and incentive systems for the academy in undertaking research coproduction, the skills, capabilities, and competencies to be able to do research coproduction well.^18^

 In essence, these features relate to the readiness of the research ecosystem for research coproduction to be part of business as usual. Arguably, there is some way to go to evolve and transform the research landscape.

## Implications

 Current structures, governance and policy frameworks tend to be organized in a way that prioritises traditional knowledge transfer or dissemination approaches. As outlined above, there are some critical features that distinguish research coproduction from approaches that conceptualize researchers and knowledge users as two distinct communities. Within research coproduction the borders of expertise between those who do research and those who use it are more permeable. To achieve this, attention at micro, meso, and macro levels is required.

 At a macro or societal level there is an increasing focus on inclusion and involvement of “non-experts” in the production of goods and services^19^ including in health; this is evidenced in the conceptual shift to viewing patients as partners in their healthcare.^8,9^ Research coproduction also aligns with calls for equity, diversity, inclusion and social justice. Within a health research context, embedded patient and public involvement and engagement has gathered real momentum to the point where some funders (for example, UK’s National Institute for Health Research, Canadian Institutes of Health Research) will not recommend funding if meaningful involvement of knowledge users in the research lifecycle is not evident. There are important implications for rebalancing the privileging of science and as such, research coproduction, as an inclusive process, including sharing power with those who are not scientists to “build a new vision of academia where academics and communities work together, create research cultures together, respect and value each other, seek and value each other’s knowledge…”^20^ (p. 3).

 At a meso level, research coproduction requires a radical shift in the academy including our belief and values system, and skills and competencies to fully embrace and enact partnership work and inclusive practices. The academy is operating within a system that is largely counterproductive to meaningful research coproduction. One example of this is in the way in which researchers are rewarded. Typical promotion routes include a measure of the quality and quantity of peer-reviewed publications, outputs that are of less value or use to knowledge users. Whilst initiatives such as the Resumé for Researcher and Innovation, and the Declaration on Research Assessment, are useful for emphasising the broader contribution of researchers and research, and researchers themselves are adding collaborations with knowledge users to their curriculum vitae,^18^ these mechanisms are far from being embedded and valued in the academy.

 The way in which research is funded requires attention. Funders are in an ideal position to accelerate research coproduction through sources that fund coproduction projects and programmes, including funding for foundational activities such as relationship building, supporting skills development through training and fellowship opportunities, and ensuring adequate resources for engaged knowledge mobilization. There are also implications for the criteria on which research coproduction funding applications are evaluated, including attention to assessing the relevance of the problem or uncertainty from a knowledge user perspective, and on judging the quality of the researcher-knowledge user partnership. Providing opportunities for knowledge users to apply for research funding would also support a shift in the power balance needed for authentic research coproduction. Expanding funding opportunities for infrastructure awards that focus on academic-knowledge user organisation collaborations would enable the development of sustained partnerships and programmes of research coproduction. Existing examples include the UK’s National Institute for Health and Care Research’s Applied Research Collaborations, and the Health Determinants Research Collaborations where knowledge user organisations are in receipt of funding. However, these types of funding opportunities are not commonplace. Perhaps the greatest potential for transformation would be in developing more opportunities for between-organisation collaborations to develop a sustainable research coproduction ecosystem and as a result, real world implementable evidence.

 The structures and process that both govern and support research also need attention for research coproduction to flourish. For example, research ethics processes typically include the need for upfront, detailed protocols of activity whereas research coproduction proposals are developmental and emerging based on partnership interactions and the roles of individuals in the projects may be both as participants and knowledge users. Ethics review systems need to be both robust and flexible to accommodate more emergent research projects and programmes.

 At a micro level, researchers’ and knowledge users’ capability and capacity to engage in meaningful research coproduction is clearly critical but an area where there is a particular gap in our understanding. As research coproduction is not aligned with any one method, a suite of competencies is required that includes relationship building and maintenance. Finely tuned communication and interpersonal skills are required as is emotional intelligence. Typically, team members learn as they are doing, however, the competencies required for research coproduction require us to reflect on both the content and delivery of research training in a more systematic way to reflect the distinctiveness of a research coproduction approach and to facilitate best practice.

## Conclusion

 In the context of the continued challenge to ensure that health and social care research results in impact, we suggest that it is time for an evolutionary shift in the research cycle. We must extend our thinking about knowledge to action, in which knowledge and action is inextricably linked through research coproduction. It is a principle-based approach driven by a set of values that extends the idea of research collaboration and engagement to one of equity focused research coproduction. Whilst coproduction and participatory research have a long history, coproduction within health care contexts and health is relatively young. We suggest that systematizing research coproduction provides both the opportunity and mechanism for co-developing evidence-informed solutions to real world challenges that are more useful, useable, and ultimately, used to reduce health inequities. We call on knowledge users and researchers to embrace this way of thinking, continue to develop the research coproduction toolbox and to share learning. By doing so, more inclusive research, which more rapidly translates to better and more equitable health and care for all will result.

## Acknowledgements

 We thank Madeline Dougherty for support in formatting the manuscript.

## Ethical issues

 Not applicable.

## Competing interests

 Authors declare that they have no competing interests.

## Funding

 IDG is a recipient of a CIHR Foundation Grant (FDN# 143237).
